# Voluntary Spatial Attention has Different Effects on Voluntary and Reflexive Saccades

**DOI:** 10.1100/tsw.2003.72

**Published:** 2003-09-15

**Authors:** Stephanie K. Seidlits, Tammie Reza, Kevin A. Briand, Anne B. Sereno

**Affiliations:** Department of Neurobiology and Anatomy and the Keck Center for the Neurobiology of Learning and Memory, University of Texas-Houston Medical School, 6431 Fannin Street, Houston, TX 77030, USA

**Keywords:** spatial attention, prosaccades, antisaccades, voluntary, reflexive

## Abstract

Although numerous studies have investigated the relationship between saccadic eye movements and spatial attention, one fundamental issue remains controversial. Some studies have suggested that spatial attention facilitates saccades, whereas others have claimed that eye movements are actually inhibited when spatial attention is engaged. However, these discrepancies may be because previous research has neglected to separate and specify the effects of attention for two distinct types of saccades, namely reflexive (stimulus-directed) and voluntary (antisaccades). The present study explored the effects of voluntary spatial attention on both voluntary and reflexive saccades. Results indicate that voluntary spatial attention has different effects on the two types of saccades. Antisaccades were always greatly facilitated following the engagement of spatial attention by symbolic cues (arrows) informing the subject where the upcoming saccade should be directed. Reflexive saccades showed little or no cueing effects and exhibited significant facilitation only when these cues were randomly intermixed with uncued trials. In addition, the present study tested the effects of fixation condition (gap, step, and overlap) on attentional modulation. Cueing effects did not vary due to fixation condition. Thus, voluntary spatial attention consistently showed different effects on voluntary and reflexive saccades, and there was no evidence in these studies that voluntary cues inhibit reflexive saccades, even in a gap paradigm.

## INTRODUCTION

Previous research involving the relationship between spatial attention and saccades has led to disagreement on the seemingly basic issue of whether or not spatial attention facilitates, inhibits, or has no effect on saccadic eye movements. The view that voluntary spatial attention facilitates saccades has been suggested by several studies (e.g., [1]). This facilitation is often interpreted as an indication that endogenous shifts of attention precede endogenous saccades[2]. Theoretical proposals such as the "premotor" theory of attention[3,4] are supportive of this view. The premotor theory asserts that by preparing an attentional shift, one automatically also prepares an oculomotor or manual response. It assumes that since the spatial maps used to code shifts of attention in space are also involved in the preparation of oculomotor responses, that preparation to shift attention inevitably leads to the preparation of a saccade to that location. And, indeed, recent imaging work suggests an overlap in some cortical regions for attending, looking, and pointing[5].

However, other studies[6] have proposed that spatial attention actually inhibits saccades. For example, these studies have reported delayed saccadic latencies in a gap task when spatial attention is activated, even when the cue accurately predicts the target location[7,8,9]. According to Fischer and colleagues, attention must first be disengaged before an eye movement can be made. Hence, even attending to the target location should delay the eye movement to the target. Further, they argue that the "gap" in a gap paradigm allows the subject to disengage spatial attention from the point of fixation before making a saccade. Hence, there is less of an attentional cost in the uncued gap paradigm. In addition, studies have reported that antisaccades are both slower and less accurate when precued to attract attention[10,11].

In addition to these two approaches, there have been a number of other proposals. For example, there are models that support the idea of a functional relationship between the two processes [12], some that dispute a physiological relationship between them [13], and others that argue against any specific attentional effects on saccades at all, either facilitatory or inhibitory [14]. In this last framework, saccadic performance is largely a function of low-level perceptual representations and the reciprocal inhibition between them. Attentional influences on eye movements are reduced to a minimal level.

These inconsistencies in the literature might be resolved if more consistent, precise definitions of spatial selective attention and saccadic eye movements were applied. Much work now supports a dissociation between voluntary and reflexive saccades. For instance, Henik et al.[15] demonstrated that patients with unilateral prefrontal lesions including the frontal eye fields showed longer latencies to the contralesional visual field when performing a voluntary saccade task, but shorter latencies to the contralesional field when performing a reflexive saccade task. Also, Machado and Rafal[16] demonstrated a dissociation in the modulation of the fixation offset effect for voluntary and reflexive saccades and suggested that this dissociation was supportive of neural activity changes in two different parts of the brain. In addition to a dissociation in overt orienting, a similar distinction between a voluntary (endogenous) and a reflexive (exogenous) process has been demonstrated with respect to covert orienting (i.e., spatial attention[17]). Drawing from this large amount of evidence suggesting that both eye movements (overt orienting) and spatial attention (covert orienting) can be subdivided into two categories, voluntary and reflexive, Sereno[18] has proposed that systematically distinguishing between voluntary and reflexive processes might clarify these issues.

A reflexive saccade is a seemingly automatic eye movement elicited by a target, such as in the prosaccade task, where the subject must make a saccade to a sudden onset stimulus occurring in an unpredictable location. Alternatively, voluntary saccades require an eye movement that is internally generated. For example, in the antisaccade task, the subject must make an eye movement to a point in the visual field opposite the stimulus, and thus suppress a reflexive prosaccade towards the stimulus.

With respect to covert orienting, or shifts of spatial attention in the absence of eye movements, a similar distinction between voluntary and reflexive processes can be made. The nature of the attentional shift is determined by the method used to direct spatial attention. In a reflexive task, a peripheral, exogenous cue (e.g., the brightening of a box) indicates the upcoming target location[12]. This is similar to reflexive prosaccades, because an actual sensory event is used to attract attention to a position in the visual field. Facilitation effects occur very rapidly, within 50–100 ms after cue onset[19]. In contrast, a voluntary attentional task involves the presentation of a symbolic cue (e.g., an arrow) at the central fixation point indicating where the upcoming target will most likely appear. Similar to reflexive attention, voluntary cueing also benefits the detection of the upcoming target. However, the task exhibits a much slower and less effective facilitation beginning only 200–300 ms after cue onset[20,21].

Sereno[22] proposed a model of attention and eye movements that included separate reflexive and voluntary components. Sereno's tonic-inhibition model of orienting asserted that separate neural systems control voluntary and reflexive saccades (involving the frontal eye fields and the prefrontal cortex, and the superior colliculus, respectively) and that the voluntary system plays a controlling role with respect to the tonic inhibition of the reflexive system. Different lines of research have suggested that the frontal eye fields play a controlling and inhibitory role with respect to the superior colliculus, involving an indirect pathway through the caudate and substantia nigra pars reticulata[23,24,25]. Voluntary control involves both the activation of voluntary processes as well as perhaps an increase in inhibition of the reflexive eye movement system. Thus, Sereno's model predicts that voluntary attention would affect voluntary and reflexive saccades differently. While voluntary attention should facilitate voluntary saccades, it may also increase inhibition of the reflexive system and hence slow reflexive saccades.

These predictions were tested in a preliminary investigation by Sereno[26]. The study investigated the effects of voluntary spatial attention in both the prosaccade and the antisaccade tasks. The cues were always valid. Subjects performed each condition (Prosaccade, Prosaccade-Cued, Antisaccade, Antisaccade-Cued) in four separate blocks counterbalanced across subjects. Results indicated that voluntary attentional cues affected voluntary and reflexive saccades differently. As expected, spatial attention facilitated voluntary saccades (Table 1). However, there was a nonsignificant trend suggesting voluntary attention inhibits reflexive saccades. The present study attempts to expand on this preliminary study in order to clarify exactly how voluntary spatial attention affects voluntary and reflexive eye movements and perhaps resolve inconsistencies in the literature[1,6,12].

**TABLE 1**
**Mean Response Times and (% Error) from Sereno[26]**

| Task | Uncued | Cued | Uncued – Cued |
|---|---|---|---|
| Antisaccade | 361 (3.2%) | 329 (0.3) | 32 ms[a] (2.9%) |
| Prosaccade | 299 (0.2%) | 314 (0.1) | –15 ms[b] (0.1%) |

[a]$p < 0.01$; [b]$p > 0.17$

It is important to resolve the issues surrounding effects of voluntary attention on reflexive and voluntary eye movements in order to better understand how covert and overt orienting mechanisms interact. The present study investigated whether voluntary attention truly has different effects on voluntary and reflexive saccades, and what conditions might modulate these effects. The first experiment attempted to replicate the original experiment by Sereno[26], using the same four blocks of trials described previously. The second experiment investigated whether mixing cued and uncued trials alters the results. The third experiment replaced previously uncued trials with a neutral cue to control for alerting effects. The fourth experiment further explored the effects of fixation condition, using neutral and directional cues.

## EXPERIMENT 1

### Methods

#### *Subjects*

Sixteen college students ranging between the ages 18 and 24 were tested as normal control subjects. All subjects had normal or corrected vision.

#### *Apparatus*

Eye movements were recorded using a high-speed infrared sensitive camera interfaced with an ISCAN RK-426 eye tracking system. Gaze position was converted to screen coordinates and displayed on a second monitor to inform the experimenter, in real time, where the subject was looking. Spatial resolution of the eye tracking system was approximately 0.5° of visual angle and temporal resolution was set at 180 samples/second. Saccades were detected when eye velocity exceeded 100°/second and ended when eye velocity fell below 12°/second.

#### *Stimuli*

Subjects placed their head on a chin rest positioned 72 cm from a computer monitor employed to display the stimuli (Sony Trinitron Multiscan sf II, 17 in.). From this distance, the monitor screen covered a visual area of 25° × 18°. The target appeared 7° to the left and right of a gray central fixation point of 0.15° projected against a black background. The target was a white square 0.2° × 0.2°.

#### *Procedure*

Subjects performed four blocks of trials, which were counterbalanced across subjects. These blocks consisted of the following conditions: Prosaccade-No Cue, Prosaccade-Cued (100% valid), Antisaccade-No Cue, Antisaccade-Cued (100% valid). Cues were always 100% predictive of saccade direction when present. In each of the four conditions, subjects received 12 practice trials and 48 experimental trials (12 for each of the four delay intervals). Practice trials were not included in the data analysis.

**Prosaccade task** — Each trial consisted of the following sequence: (1) a blank screen (black background) with a gray central fixation point presented for 800 ms; (2) a variable delay of 400, 600, 800, or 1000 ms (to match the duration of the cue presentation, see below); (3) presentation of the target accompanied by a brief audible tone. This target screen was terminated by either an eye movement to the target (4), or by a failure to respond within 1000 ms of target onset. Following either of these outcomes, a blank screen was presented for 500 ms and then the sequence was repeated (see Fig. 1). All trials were presented in the overlap condition, where the cue or fixation point remained on the screen when the target appeared. For the prosaccade task, correct saccades were defined as saccades directed towards the target that terminated within 4.4° of the target's position. All other saccades were classified as errors.

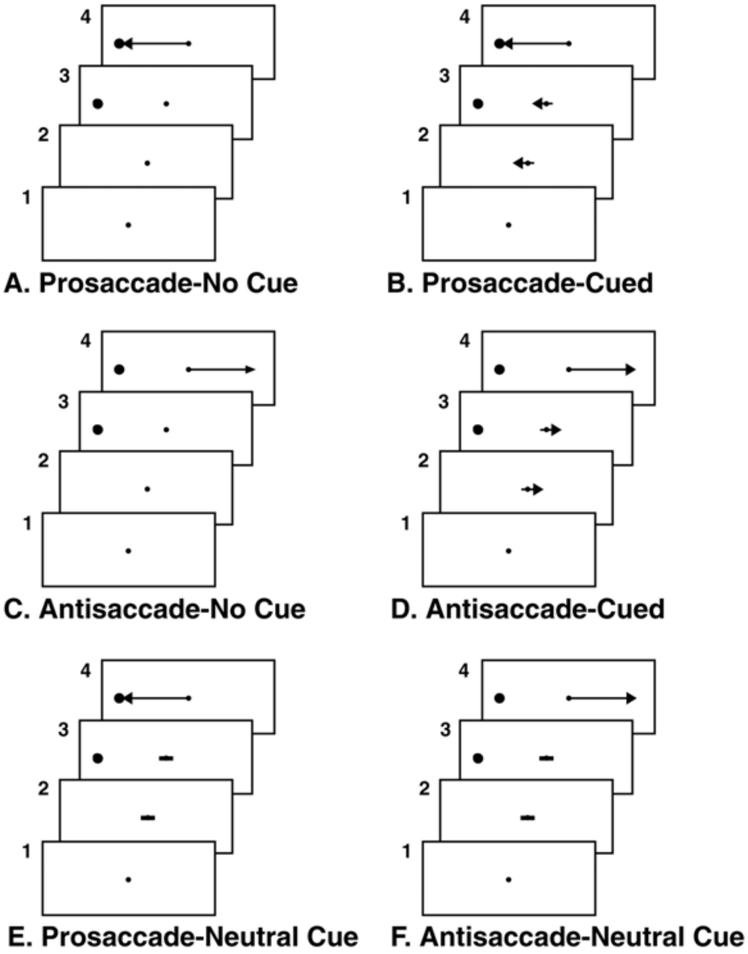

**FIGURE 1.** Prosaccade and Antisaccade Tasks. Schematic representation of a trial in the prosaccade task with no cue (panel A), prosaccade task with cue (panel B), antisaccade task with no cue (panel C), and antisaccade task with cue (panel D), prosaccade task with neutral cue (panel E), and antisaccade task with neutral cue (panel F). Each trial began with (1) the presentation of a *fixation screen* (a blank screen with a gray central fixation point) presented for 800 ms, followed by (2) a *variable delay* with either a fixation point (tasks with no cue, panels A and C), an arrow predictive of the upcoming eye movement (tasks with cue, panels B and D), or a neutral cue (panels E and F); then (3) presentation of the *target* accompanied by a brief audible tone, (4) the long arrow illustrates the *correct eye movement response* for the trial.

**Antisaccade task** — The antisaccade task was identical to the prosaccade task, except that the subject was required to make an eye movement in the direction opposite the target stimulus. Correct responses had to be within 4.4° of the screen position directly opposite the target location. All other responses, in particular saccades to the target itself, were counted as errors.

**Voluntary attention** — Voluntary spatial attention was manipulated in both the prosaccade and antisaccade tasks by presentation of a gray arrow cue at the central fixation point prior to the target presentation, which always validly indicated the direction of the upcoming eye movement. Thus for prosaccades, the arrow pointed towards the position where the target would appear, and for antisaccades it pointed towards the location opposite the target. There was a delay interval of variable duration between the onset of cue and target to prevent predictive saccades. The delay intervals were either 400, 600, 800, or 1000 ms and varied randomly from trial to trial (note that these matched the effective durations of the fixation periods in the no cue conditions described above). Subjects were required to wait until the target appeared prior to making a saccade. A saccade made before the target resulted in a time-out beep and aborted the trial, which would be presented again later. The frequency of these anticipatory responses was recorded for later analysis. Subjects were instructed to make a correct saccade as quickly as possible when the target appeared. Correct saccades were saccades in the same direction as the arrow cue with a proper amplitude as described above.

These four conditions were similar to the Sereno[26] procedure with the main difference being that the earlier study had used only two delay periods instead of four.

## Results

### *Response Times*

In this experiment as in all the remaining ones, mean response times for correct trials were calculated after first excluding too fast (<100 ms) or too slow (>700 ms) responses (2.9% of all trials). Mean response times were analyzed using a three-way factorial ANOVA including the following variables: Task (Prosaccade vs. Antisaccade), Cue (Cued vs. No Cue), and Delay Interval (400, 600, 800, or 1000 ms).

There was a main effect of Task, with antisaccades (329 ms) having slower response times than the prosaccades (258 ms) ($F_{1,15} = 49.2$, $p < 0.0001$). There was also a main effect of Cue with response times being faster in the Cued condition (278 ms) vs. the No Cue condition (309 ms) ($F_{1,15} = 18.9$, $p < 0.0006$). Delay Interval was not significant ($F_{3,45} = 1.15$, NS). Task by Cue had a significant interaction ($F_{1,15} = 11.2$, $p < 0.005$) with larger cueing effects for antisaccades than prosaccades (56 and 6 ms, respectively). In addition, there was a significant interaction between Task and Delay ($F_{3,45} = 5.54$, $p < 0.003$) with the prosaccade-antisaccade difference being reduced at longer delay intervals (differences of 91, 73, 54, and 67 ms for the shortest to longest delay periods). Finally, the interaction of Delay Interval by Task by Cue was not significant ($F < 1$, NS).

### *Anticipations*

Saccades executed during the delay period prior to the target onset, reflect anticipation of the upcoming stimulus event. These trials were counted and then aborted and randomly represented later in the session. Hence, they were not included in the reaction time or error rate data. However, the raw number of anticipatory responses made by each subject was used for this analysis (Fig. 2). If these anticipations occurred more frequently on cued trials, it is likely that some anticipatory

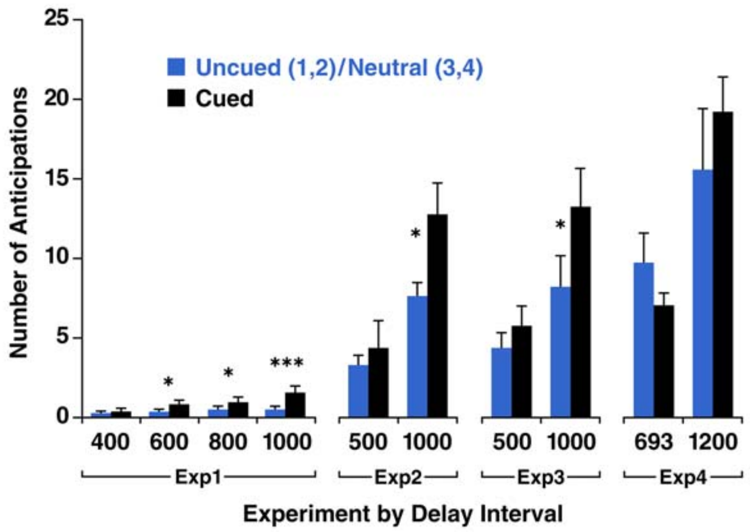

**FIGURE 2.** Anticipatory responses, Experiment 1 and all others. Anticipations before the target onset for cued, no cue, or neutral trials at each delay interval for all four experiments. Generally, more anticipations were made at the longer delay intervals on cued trials. Asterisks (*) indicate the degree of significance (i.e., one asterisk indicates $p < 0.05$, two $p < 0.01$, three $p < 0.001$, four $p < 0.0001$). These same markings are used in all figures. Exp1, Experiment 1; Exp2, Experiment 2; Exp3, Experiment 3; Exp4, Experiment 4.

responses may have gone undetected (i.e., latencies greater than 90 ms) and contributed to faster overall performance on cued trials. Hence, the cueing effects we observed may have nothing to do with facilitation due to voluntary attention per se. Therefore, it is important to verify that a performance difference between the cued and uncued conditions is not contaminated by the presence of anticipatory responses on cued trials. Accordingly, anticipatory responses were analyzed using a three-way factorial ANOVA including Task (Prosaccade vs. Antisaccade), Cue (Cued vs. No Cue), and Delay Interval (400, 600, 800, or 1000 ms) as factors.

Anticipations were more likely to occur as delay interval increased ($F_{3,45} = 4.90$, $p < 0.005$). They were also more likely to occur on cued trials rather than trials with no cue ($F_{1,15} = 4.56$, $p < 0.05$). There was also a significant interaction of Delay by Cue ($F_{3,45} = 3.57$, $p < 0.03$). This interaction was due to an increase in anticipations on cued trials relative to uncued trials at the longer delay intervals ($p < 0.05$, $p < 0.06$, and $p < 0.0001$ for 600-, 800-, and 1000-ms delays, respectively) but not at the shortest delay ($p > 0.58$). Since greater anticipation rates could possibly affect the response times and mask or contaminate purely attentional effects, the response times and error data from only the 400-ms (short delay) interval were analyzed separately to examine the influence of voluntary attention.

## Response Times — 400-ms Delay Interval

Mean response times from the 400-ms delay condition were analyzed by a two-way ANOVA with Task (Prosaccade vs. Antisaccade) and Cue (Cued vs. Uncued) as factors (see Fig. 3). There were main effects of Task ($F_{1,15} = 50.3$, $p < 0.0001$) and Cue ($F_{1,15} = 6.00$, $p < 0.03$). There was also a significant interaction of Task by Cue ($F_{1,15} = 12.5$, $p < 0.003$). Planned comparisons using the MSE from the Task by Cue interaction (MSE = 1213.753) showed that the cueing effect for prosaccades was not significant (0 ms, $F < 1$, $p > 0.98$). However, the cueing effect was highly significant for antisaccades (62 ms, $F_{1,15} = 24.9$, $p < 0.0002$) (Fig. 3).

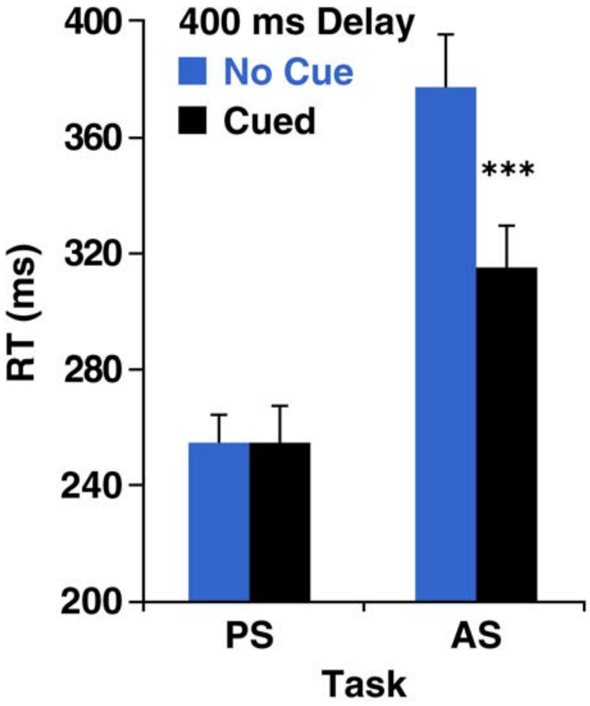

**FIGURE 3.** Response times, Experiment 1. Mean response times for Experiment 1 (400-ms delay only) on cued and no cue trials for prosaccade and antisaccade tasks. Cues facilitated antisaccades but not prosaccades. PS, prosaccade task; AS, antisaccade task.

## *Error Rates*

Analysis of error rates from the 400-ms delay interval used a two-way ANOVA with factors of Task and Cue at just the short delay. This analysis showed significant effects of Task ($F_{1,15} = 15.8$, $p < 0.002$; more errors for the AS task) and Cue ($F_{1,15} = 5.96$, $p < 0.03$; fewer errors on cued trials). There was also a significant interaction of Task by Cue ($F_{1,15} = 14.0$, $p < 0.002$). Planned comparisons (MSE = 58.907) showed that cues reduced errors for antisaccades ($F_{1,15} = 37.5$, $p < 0.00002$), but not for prosaccades ($F_{1,15} < 1$, NS) (Fig. 4). Therefore, voluntary attentional cues appear to significantly aid accuracy in antisaccade performance while having little affect on prosaccades.

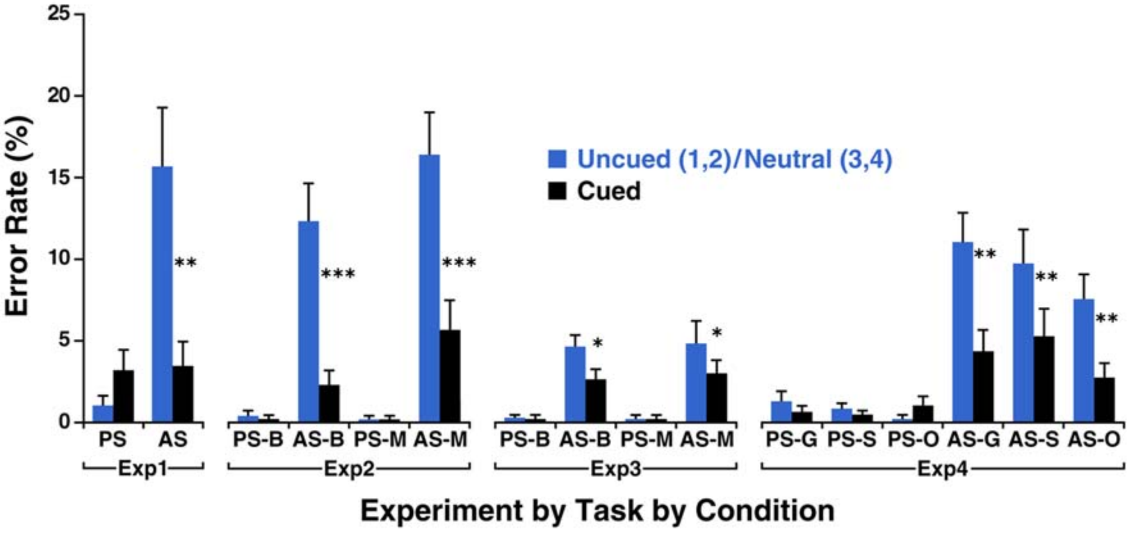

**FIGURE 4.** Error rates, Experiment 1 and all others. Error rates for cued versus no cue or neutral cue trials for all tasks in each experiment for the shortest delay only. Errors were most likely to occur in the antisaccade task on trials with no directional cue. Exp1, Experiment 1; Exp2, Experiment 2; Exp3, Experiment 3; Exp4, Experiment 4; PS, prosaccade task; AS, antisaccade task; B, blocked presentation; M, mixed presentation; G, gap condition; S, step condition; O, overlap condition.

## Discussion — Experiment 1

The first experiment provides strong evidence that voluntary attentional cues have different effects on voluntary and reflexive eye movements. Antisaccades were faster and more accurate when preceded by cues, but reflexive eye movements were unaffected. However, while results indicated that spatial attention does not have uniform effects on voluntary and reflexive eye movements, there was no evidence for inhibition of reflexive eye movements by voluntary spatial attention as seen in previous reports ([6]; see also Sereno[26] for a trend supportive of such an inhibitory effect).

## EXPERIMENT 2 — BLOCKED VS. MIXED ATTENTION CUES

Experiment 2 further investigated the effects of cueing conditions. Previous accounts had suggested that voluntary spatial attention might actually inhibit reflexive eye movements (e.g., [6,18,26]). In contrast, Experiment 1 revealed no effect of voluntary cues on reflexive saccades. It is possible that subjects ignored the cue in the prosaccade task in Experiment 1, and simply responded to the target stimulus. This would account for the absence of any effect of cues (facilitatory or inhibitory) in the reflexive saccade task. Perhaps, the presentation of cues in separate blocks of trials might have increased this possibility. In 1980, Posner et al.[27] showed that spatial cues only lead to cueing effects in a spatial orienting paradigm when they are presented under mixed conditions (i.e., valid and invalid cues mixed). Whereas this clearly does not explain the data for voluntary saccades (which were facilitated by the voluntary spatial cues), it may explain why there was no effect seen for reflexive saccades. It is possible that the cues, despite their validity, were rendered less effective because they were presented in a separate block of trials.

In order to make the cues more salient, cued and uncued trials were mixed within the same block. For comparison, subjects also performed the cued and uncued conditions under blocked presentation.

Two questions were addressed in this experiment: (1) is there a larger cueing effect in the mixed condition (an increased influence of the cues) and (2) if there is a larger cueing effect under mixed presentation, then is it the same for the prosaccade and the antisaccade task?

## Methods

### Subjects

Sixteen college students, who did not participate in any previous experiments, ranging between the ages 18 and 24 were tested as normal control subjects. All subjects had normal or corrected vision.

### Stimuli and Procedure

Methods were identical to Experiment 1, with three exceptions. Cues were presented either as in Experiment 1 (namely, blocked cue presentation) or else were presented in a mixed manner. Furthermore, there were only two delay intervals between cue and target (500 and 1000 ms) instead of four. Finally, instead of using a velocity criterion to detect saccades as in Experiment 1, a location-based criterion was used in this and subsequent experiments. A saccade was assumed to have been initiated when point of gaze moved beyond 2.4° from fixation.

Subjects performed one set of eye movement trials for each of six different conditions, for a total of six sets of trials. There were four blocked conditions and two mixed. The order of the sets was counterbalanced across subjects (the four blocked conditions were presented before the two mixed, or vice versa). The six conditions that were tested included the following: Prosaccade-No Cue, Prosaccade-Cued, Antisaccade-No Cue, Antisaccade-Cued, Prosaccade-Mixed (50% no cue, 50% cued), and Antisaccade-Mixed (50% no cue, 50% cued) (see Fig. 1). Cues were always 100% predictive of saccade direction when present. The blocked cue–type conditions replicate the conditions in Experiment 1, whereas the mixed conditions were intended to increase the salience of the cues. In each of the four blocked conditions, subjects received 12 practice trials and 60 experimental trials (30 at each delay interval). In each of the two mixed conditions, subjects received 12 practice trials and 120 experimental trials. Practice trials were not included in the data analysis.

## Results

### Response Times

Data trimming resulted in exclusion of 2.7% of all trials (same criteria as for Experiment 1). Mean response times of the remaining correct responses were analyzed using a four-way factorial ANOVA including the following four variables: Task (Prosaccade vs. Antisaccade), Cue (Cued vs. Uncued), Condition (Blocked vs. Mixed), and Delay Interval (500 ms-Short vs. 1000 ms-Long).

There was a main effect of Task, with the antisaccade task having slower response times than the prosaccade task (317 and 247 ms, respectively; $F_{1,15} = 68.3$, $p < 0.0001$). There was a main effect of Cue with response times being faster in the Cued condition (265 ms) vs. the Uncued condition (300 ms) ($F_{1,15} = 38.9$, $p < 0.0001$). Task by Cue showed a significant interaction ($F_{1,15} = 21.0$, $p < 0.004$) with a larger cueing effect for antisaccades (52 ms) than for prosaccades (19 ms). There was a main effect of Delay Interval (Short vs. Long) ($F_{1,15} = 7.25$, $p < 0.02$) with faster responses at the longer delay. There was no main effect of Condition (Blocked vs. Mixed) ($F_{1,15} < 1$, NS) nor a significant interaction between Condition and Cue ($F_{1,15} = 2.40$, $p > 0.14$). However, there was a trend towards a larger cueing effect in mixed (41 ms) vs. blocked presentation (29 ms). Finally, there was a marginally significant interaction of Delay Interval by Condition by Task by Cue ($F_{1,15} = 3.67$, $p < 0.08$).

## Anticipations

As in Experiment 1, anticipations were analyzed to check whether these increased with delay period. Saccades prior to target onset, or anticipations, were more likely to occur at the 1000-ms long delay interval ($F_{1,15} = 16.6$, $p < 0.001$) and following cues ($F_{1,15} = 7.14$, $p < 0.02$). There was also a significant interaction of Delay and Cue ($F_{1,15} = 8.94$, $p < 0.01$). There was an increase in anticipations on cued trials relative to uncued trials at the long ($p < 0.02$) but not at the short delay interval ($p > 0.61$) (Fig. 2). Since greater anticipation rates at the long delay interval could falsely mimic an attentional benefit by lowering response times, only response times and errors from the 500-ms delay interval were analyzed.

## Response Times — 500-ms Delay Interval

Data from the short condition were analyzed by a three-way ANOVA with Task (Prosaccade vs. Antisaccade), Cue (Cued vs. Uncued), and Condition (Blocked vs. Mixed) as factors.

There were main effects of Task ($F_{1,15} = 68.2$, $p < 0.0001$) with antisaccades (322 ms) slower than prosaccades (249 ms), as well as of Cue ($F_{1,15} = 50.32$, $p < 0.001$) with Cued (268 ms) faster than Uncued (303 ms) trials. There was no main effect of Condition ($F_{1,15} = 1.12$, $p > 0.30$, NS). Task by Cue showed a significant interaction ($F_{1,15} = 23.0$, $p < 0.0002$) with antisaccades (52 ms) having a larger cueing effect than prosaccades (18 ms). There was also marginally significant interaction of Condition by Cue ($F_{1,15} = 3.99$, $p < 0.07$) with cueing effects larger in the mixed (43 ms) than in the blocked condition (29 ms). There was no significant interaction of Condition by Task by Cue ($F_{1,15} < 1$, NS).

Planned contrasts were carried out on the cueing effects for the four possible combinations of Task by Condition. Planned comparisons showed that the simple cueing effects were marginal in the prosaccade blocked condition ($F_{1,15} = 3.29$, $p < 0.09$) and significant in the remaining three conditions: prosaccade mixed ($F_{1,15} = 27.9$, $p < 0.0001$), antisaccade blocked ($F_{1,15} = 40.0$, $p < 0.00001$), and antisaccade mixed ($F_{1,15} = 142$, $p < 0.000001$) (Fig. 5). Additional planned contrasts confirmed that cueing effects were greater for antisaccades than for prosaccades, both in the blocked condition ($F_{1,15} = 20.3$, $p < 0.0004$) and the mixed ($F_{1,15} = 25.2$, $p < 0.0002$). Finally, cueing effects were marginally increased in the mixed over the blocked presentation for antisaccades ($F_{1,15} = 3.25$, $p < 0.10$), but not significantly for prosaccades ($F_{1,15} = 2.45$, $p < 0.14$).

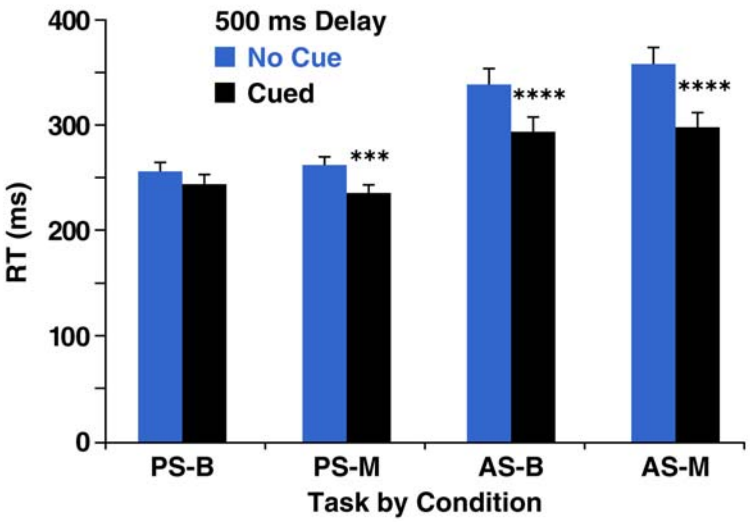

**FIGURE 5.** Response times, Experiment 2. Mean response times for Experiment 2 (500-ms delay only) for cued vs. no cue trials for prosaccade and antisaccade tasks in mixed and blocked presentations. Cueing effects were significant for all antisaccade trials, but for prosaccade trials only in the mixed presentation. PS, prosaccade task; AS, antisaccade task; B, blocked presentation; M, mixed presentation.

### *Error Rates*

A three-way ANOVA analysis (using Condition, Task, and Cue) of error rates from the 500-ms delay interval showed significant effects of Task ($F_{1,15}$ = 42.3, $p < 0.0001$) with more errors for the antisaccade task (9.2%) than the prosaccade task (0.15%), and Cue ($F_{1,15}$ = 31.15, p < 0.0001) with fewer errors on cued trials (2.0%) than uncued trials (7.3%). There was a marginal influence of blocked vs. mixed cue presentation on cueing effects ($F_{1,15}$ = 3.32, $p < 0.09$), with more errors under mixed presentation. There was a significant interaction of Condition by Task ($F_{1,15}$ = 4.77, $p < 0.05$) with an increase in errors in mixed presentation for antisaccades (+3.7%) but not for prosaccades (–0.3%). Finally, there was a significant interaction of Task by Cue ($F_{1,15}$ = 26.0, $p < 0.0001$) with a reduction in errors on cued trials for antisaccades (–10.4%) but not for prosaccades (–0.1%) (Fig. 4). Planned comparisons showed that cues did not reduce errors for prosaccades under either mixed or blocked presentation ($F_{1,15} < 1$ for both), but that they did reduce errors for antisaccades ($p < 0.0001$ for both mixed and blocked presentation).

## Discussion — Experiment 2

Addressing the possibility that subjects might ignore the cues in the prosaccade task, Experiment 2 made these cues more salient by mixing cued and uncued trials. This mixed condition marginally increased the cueing effect for antisaccades in both response time and error analyses. Notably, voluntary cues did have significant benefits on reflexive saccades in the mixed presentation and even a trend towards significance (marginally, $p < 0.09$) in the blocked condition. This latter effect was not found in Experiment 1 nor reported previously[6,26]. Under mixed cue presentation, there was significant facilitation of prosaccades. We found no evidence for an inhibitory influence of voluntary attentional cues on prosaccades.

These findings demonstrate that voluntary attention benefits voluntary saccades to a greater extent than reflexive saccades. The results also show that the effect of voluntary attentional cues on prosaccades depends on whether the different cueing conditions are blocked or mixed; facilitatory effects are increased when attentional cues are made more salient under mixed conditions.

## EXPERIMENT 3 — NEUTRAL CUE

In the third experiment, we attempted to control for alerting properties of the spatial cues. It should be noted that the attentional cues we utilized provided not only information concerning where an upcoming eye movement was to be directed, but also when it should be initiated. This is because on cued trials, the appearance of the arrow acts as a signal that allows subjects to prepare for the appearance of the subsequent target. Even with a variable cue-target interval, this may help subjects to prepare an eye movement in the cued condition, whereas in the uncued condition there is no such temporal cue.

Thus, the cues have alerting properties that may facilitate performance independent of any effects due to allocation of spatial attention per se. It is possible that better performance on cued vs. uncued trials in the reflexive saccade task may be partially due to such alerting effects. In order to equalize possible alerting properties of the voluntary cues, Experiment 3 presented spatially noninformative, neutral cues, rather than no cue, on uncued trials.

### Methods

#### *Subjects*

Sixteen college students ranging between the ages 18 and 24 were tested as normal control subjects. All subjects had normal or corrected vision.

#### *Stimuli and Procedure*

Prosaccade and antisaccade tasks, as well as the delay interval between the cue and target, were identical to Experiment 2. Subjects performed one set of eye movement trials for each of six different conditions identical to those in Experiment 2, except that a neutral cue rather than a blank screen was presented on uncued trials. Voluntary spatial attention was manipulated in both the prosaccade and antisaccade tasks by presentation of a gray arrow cue at the central fixation point prior to the target presentation, which always validly indicated the direction of the upcoming eye movement. On uncued trials, a gray, spatially noninformative, neutral cue of equal luminance appeared at the central fixation point (see Fig. 1). Thus, both the cued and uncued (neutral) trials presented subjects with a stimulus prior to target presentation that could serve as a temporal cue.

### Results

#### *Response Times*

Exclusion of fast and slow responses eliminated 3.1% of all trials. Mean correct response times were analyzed using a four-way factorial ANOVA including the following four variables: Task (Prosaccade vs. Antisaccade), Cue (Cue vs. Neutral Cue), Condition (Blocked vs. Mixed), and Delay Interval (500 ms-Short vs. 1000 ms-Long).

There were main effects of Task ($F_{1,15}$ = 86.0, $p < 0.0001$), with antisaccades (322 ms) being slower than prosaccades (253 ms); Cue ($F_{1,15}$ = 28.0, $p < 0.0001$), with response times faster in the cued (275 ms) than neutrally cued (300 ms) trials; and Condition ($F_{1,15}$ = 7.82, $p < 0.02$), with response times slower in the mixed (294 ms) than the blocked (281 ms) condition. The effect of Delay Interval (500 ms-Short vs. 1000 ms-Long) was marginal ($F_{1,15}$ = 3.84, $p < 0.7$), with faster response times at long delay (284 ms) vs. the short (291 ms).

There was an interaction between Task and Cue ($F_{1,15}$ = 11.39, $p < 0.005$), with the cueing effect greater for antisaccades (39 ms) than for prosaccades (10 ms). There was also a significant interaction of Condition by Cue ($F_{1,15}$ = 5.09, $p < 0.04$), with cueing effects larger under mixed (38 ms) than blocked presentation (19 ms). In addition, there were some marginal interactions: Delay Interval by Condition ($F_{1,15}$ = 3.49, $p < 0.09$), with a greater effect of Blocked/Mixed at short delay); Condition by Task by Cue ($F_{1,15}$ = 3.11, $p < 0.10$); and finally Delay Interval by Condition by Task by Cue ($F_{1,15}$ = 4.05, $p < 0.07$).

## Anticipations

Anticipations were more likely to occur during the 1000-ms long delay interval ($F_{1,15}$ = 28.6, $p <$ 0.0001) and following cues ($F_{1,15}$ = 7.21, $p < 0.03$). The interaction between Delay and Cue was also significant ($F_{1,15}$ = 4.77, $p < 0.05$). As was the case in the previous experiment, cues increased anticipations only at the long ($p < 0.05$) and not the short delay interval ($p > 0.53$) (Fig. 2). Accordingly, as in Experiment 1, only response times and errors from the 500-ms short delay interval were analyzed.

## Response Times — 500-ms Delay Interval

Data in the short condition were analyzed using a three-way ANOVA with task (Prosaccade vs. Antisaccade), Cue (Cued vs. Neutral Cue), and Condition (Blocked vs. Mixed) as factors.

There was a main effect of task ($F_{1,15}$ = 75.2, $p < 0.0001$) where antisaccade tasks showed a larger cueing effect than prosaccade tasks. There was also a significant effect of Cue ($F_{1,15}$ = 30.0, $p <$ 0.0001); Task by Cue ($F_{1,15}$ = 9.17, $p < 0.009$), with larger cueing effects for antisaccades (39.5 ms) than prosaccades (14.4 ms); and Condition ($F_{1,15}$ = 9.04, $p < 0.009$). The interaction of Condition by Cue was marginal ($F_{1,15}$ = 3.05, $p < 0.11$), whereas the interaction of Condition by Task by Cue was significant ($F_{1,15}$ = 4.93, $p = 0.05$).

Planned comparisons showed that the cueing effect in the blocked, prosaccade condition (1 ms) was not significant (F < 1, NS). However the mixed-prosaccade ($F_{1,15}$ = 14.3, $p < 0.002$), blocked-antisaccade ($F_{1,15}$ =32.2, $p < 0.0001$), and mixed-antisaccade ($F_{1,15}$ =24.3, $p < 0.0004$) conditions each showed significant cueing effects (Fig. 6). Additional comparisons showed larger cueing effects for antisaccades than prosaccades under blocked presentation ($F_{1,15}$ = 15.6, $p < 0.002$), but not mixed presentation (F < 1, NS). In addition, the mixed presentation resulted in larger cueing effects than the blocked presentation for prosaccades ($F_{1,15}$ = 6.82, $p < 0.02$) but not for antisaccades (F < 1, NS).

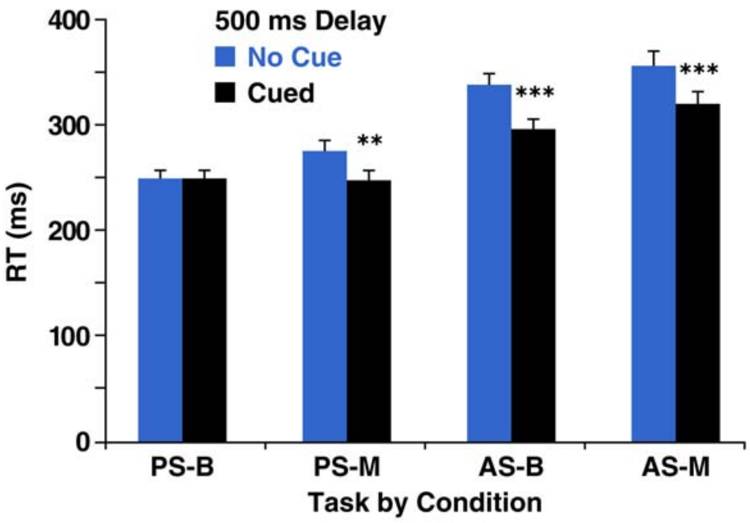

**FIGURE 6.** Response times, Experiment 3. Mean response times for Experiment 3 (500-ms delay only) for cued vs. neutral trials for prosaccade and antisaccade tasks in mixed and blocked presentations. Cueing effects were significant for all antisaccade trials, but for prosaccade trials only in the mixed presentation. PS, prosaccade task; AS, antisaccade task.

### *Error Rates*

Three-way ANOVA analysis (Condition, Task, and Cue) displayed significant main effects of Task ($F_{1,15}$ = 39.3, $p < 0.0001$) with more errors for antisaccades (3.7%) than saccades (0.27%), and Cue ($F_{1,15}$ = 6.46, $p < 0.03$) with fewer errors on cued (1.5%) than neutral (2.5%) trials. Condition was not significant (F < 1, NS). The only significant interaction was between Task and Cue ($F_{1,15}$ = 5.29, $p < 0.04$) with larger cueing effects for antisaccades (1.9%) than for prosaccades (0.0%). Planned contrasts confirmed that there was no cueing effect for prosaccades under either mixed or blocked presentation (F < 1, NS), but that cues reduced antisaccade errors under both mixed and blocked presentation ($p < 0.03$ for both) (Fig. 4).

### Discussion — Experiment 3

When spatial cues were presented under blocked conditions, voluntary attention facilitated and reduced errors for antisaccades. Cues affected reflexive saccades by shortening latencies under mixed presentation, but did not reduce errors. No cueing effects were seen on reflexive saccades presented in the blocked condition. Therefore, spatially noninformative, neutral cues eliminated the influence of voluntary cues on reflexive saccades in a blocked condition. This suggests that the marginal facilitatory effect observed for prosaccades in Experiment 2 was due not to the allocation of voluntary spatial attention per se, but rather to alerting effects of the cues. As for the mixed condition, when the cues were made more salient by mixing cued and neutral cued trials, there was facilitation of both reflexive and voluntary saccades, with significantly larger cueing effects for voluntary saccades.

## EXPERIMENT 4 — GAP, STEP, AND OVERLAP PARADIGMS

Fischer and Breitmeyer[6] asserted that not only did facilitation not occur with cueing for the prosaccade, but that inhibition actually occurs in the gap condition. Therefore, since the previous three experiments utilized the overlap paradigm, the fourth attempted to see if the gap condition might change the effects of cueing in the prosaccade task. Thus, the gap, step, and overlap paradigms were presented intermixed in four blocks of trials: Prosaccade-Cued, Prosaccade-Neutral, Antisaccade-Cued, and Antisaccade-Neutral.

## Methods

### Subjects

Sixteen naive subjects ranging from ages 19 to 23 were tested. All had normal or corrected vision.

### Stimuli and Procedure

Prosaccade and antisaccade tasks, as well as cueing procedures, were identical to Experiment 3. A different monitor (17 in. MAC Innovision) was used in this experiment. Two slightly longer delay periods were presented (693 and 1200 ms). Subjects were tested in four blocks of 180 trials: Prosaccade–Cued, Prosaccade–Neutral Cue, Antisaccade–Cued, Antisaccade–Neutral Cue (see Fig. 1). Within each block, counterbalanced across subjects, gap, step, and overlap trials were randomly intermixed.

## Results

### Response Times

Mean response times for correct trials were calculated after trimming the data to exclude unusually fast (<100 ms) or slow (>700 ms) responses (2.8% of total trials). A four-way factorial ANOVA was used to analyze the means using the following variables: Task (Prosaccade vs. Antisaccade), Cue (Cued vs. Neutral), Fixation Condition (Gap, Step, or Overlap), and Delay Interval (693 vs. 1200 ms).

All of the main effects were significant. The main effect of Task ($F_{1,15} = 176$, $p < 0.0001$) showed that the antisaccade task (288 ms) had slower response times than the prosaccade task (222 ms). Response times were also faster in the cued condition (239 ms) vs. the neutral condition (271 ms) ($F_{1,15} = 40.2$, $p < 0.0001$). Fixation Condition was also significant ($F_{1,15} = 103$, $p < 0.0001$), with the gap (232 ms) being the fastest, followed by step (252 ms), then overlap (281 ms). For Delay Interval ($F_{1,15} = 20.9$, $p < 0.0004$), the short (693 ms) interval elicited slower response times (261 vs. 249 ms). Task by Cue also had a significant interaction ($F_{1,15} = 18.3$, $p < 0.0007$), with the cue having larger effects on the antisaccade task (55 ms) than the prosaccade task (10 ms). All other interactions were nonsignificant.

### Anticipations

Anticipations were analyzed using a three-way factorial ANOVA including Delay Interval (693 vs. 1200 ms), Task (Prosaccade vs. Antisaccade), and Cue (Cued vs. Neutral). Unfortunately, the data for one subject's anticipations were lost and thus could not be included. ANOVA analysis revealed that anticipations were more likely to occur at the long Delay Interval than the short ($F_{1,15} = 23.5$, $p <$ 0.0003). No other main effects were significant. However, the interaction of Delay Interval by Cue was very close to significance ($F_{1,15} = 4.33$, $p < 0.06$). Planned comparisons revealed that for both delay periods the difference between cued and neutral trials was not significant (Long - $F_{1,15} = 1.63$, $p$

< 0.3; Short - $F_{1,15} = 1.19$, $p < 0.25$) (Fig. 2). Nevertheless, in keeping with the methods used in Experiments 1–3, the response times and error rates were analyzed just for the short delay to reduce any chance that anticipation effects might falsely appear as attentional benefits.

## Response Times — 693-ms Delay Interval

Mean response times for the short Delay Interval were analyzed using a three-way ANOVA with Task (Prosaccade vs. Antisaccade), Cue (Cued vs. Neutral), and Fixation Condition (Gap, Step, or Overlap) as factors. All of the main effects were significant. The subjects were again slower to respond on the antisaccade task (295 ms) than the prosaccade task (227 ms) ($F_{1,15} = 131.16$, $p < 0.0001$). Also, they were faster on cued (242 ms) than on neutral trials (279 ms) ($F_{1,15} = 36.84$, $p < 0.0001$). Fixation Condition showed that subjects were fastest in the gap condition (238 ms), followed by the step (257 ms), with overlap being the slowest (289 ms) ($F_{1,15} = 40.17$, $p < 0.0001$). There was a significant interaction of Task by Cue ($F_{1,15} = 12.6$, $p < 0.003$) demonstrating a greater facilitation of cueing in the antisaccade task (59 ms) relative to the prosaccade task (14 ms). There was no significant interaction of Task by Cue by Fixation Condition.

Planned comparisons showed that cueing effects were significant in the antisaccade task for gap (60.2 ms) ($F_{1,15} = 38.3$, $p < 0.00002$), step (45.9 ms) ($F_{1,15} = 22.3$, $p < 0.0003$), and overlap (71.5 ms) ($F_{1,15} = 54.0$, $p < 0.00001$) (all showed significant facilitation due to the presence of a cue). None of the cueing effects were significant in the prosaccade task for gap (15.8 ms) ($F_{1,15} = 2.62$, $p > 0.14$), step (10.3 ms) ($F_{1,15} = 1.11$, $p > 0.29$), or overlap (13.7 ms) ($F_{1,15} = 1.97$, $p > 0.19$) (Fig. 7).

Additional planned contrasts confirmed a significant difference between the cueing effect for prosaccades and antisaccades in the gap (44.4 ms) ($F_{1,15} = 10.42$, $p < 0.006$), step (35.7 ms) ($F_{1,15} = 6.72$, $p < 0.03$), and overlap (57.8 ms) ($F_{1,15} = 17.66$, $p < 0.0008$) conditions.

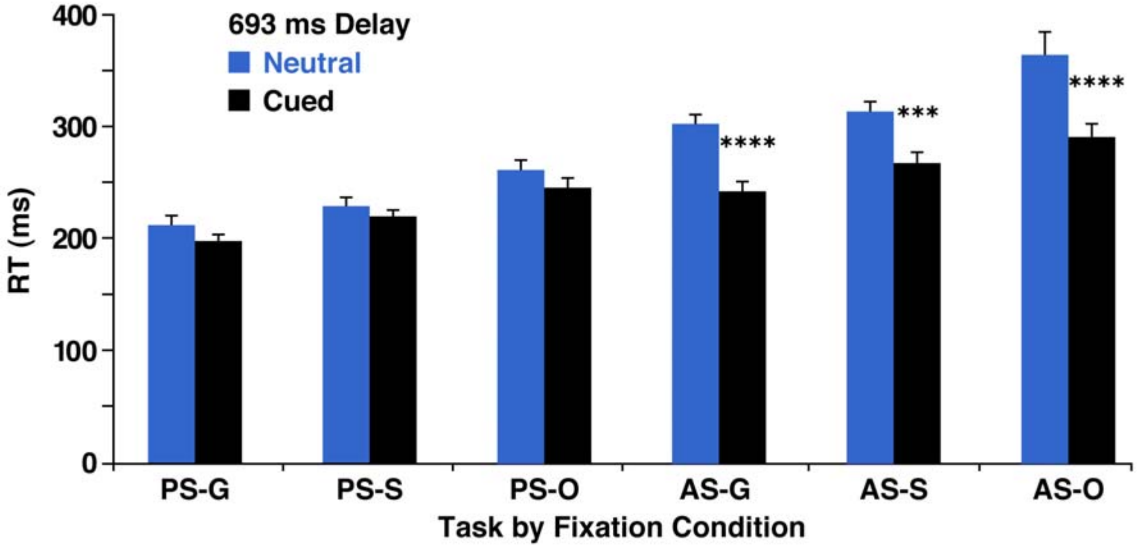

**FIGURE 7.** Response times, Experiment 4. Mean response times for Experiment 4 (693-ms delay only). Cueing effects (cued vs. neutral trials) are compared for prosaccade and antisaccade task performed with all three fixation conditions. Fixation condition had no significant effects on cueing effects. PS, prosaccade task; AS, antisaccade task; G, gap condition; S, step condition; O, overlap condition.

### *Error Rates for Short Delay Interval (693 ms)*

Three-way ANOVA analyses (Task, Cue, Fixation Condition) of errors showed that Task was significant ($F_{1,15} = 36.5$, $p < 0.0001$) with more errors made in the antisaccade than prosaccade (6.77 vs. 0.73%). Cueing was also significant ($F_{1,15} = 12.9$, $p < 0.003$) with less errors made when the cue was presented (5.10 vs. 2.0%). Task by Cue displayed a significant interaction ($F_{1,15} = 13.6$, $p < 0.003$) as well, with more errors being made on the antisaccade task with the neutral cue (9.4%) than the directional cue (4.1%). The voluntary cue did not seem to affect error rates for the prosaccade task (neutral – 0.76% vs. cued – 0.69%) (F < 1, NS) (Fig. 4). Planned comparisons showed that cueing significantly reduced errors in the antisaccade task ($F_{1,15} = 9.33$, $p < 0.01$), but had no effect in the prosaccade task (F < 1).

### Experiment 4 — Discussion

In all three paradigms (gap, step, and overlap), voluntary attention significantly facilitated antisaccades ($p < 0.001$). We did not see any evidence that the cues inhibited any eye movements, as Fischer and Breitmeyer[6] asserted. Even in the gap paradigm, the presence of cues did not inhibit latencies, and actually all fixation conditions displayed some degree of facilitation from the cue (albeit nonsignificant). Although subjects were fastest on the gap paradigm, probably due to a disengagement from the fixation point prior to the saccade, the voluntary attentional cue did not inhibit saccadic latencies. In fact, the smallest attentional affects were seen in the intermediate fixation condition (i.e., the step paradigm). Thus, the effects of voluntary spatial attention on both types of saccades appear to be relatively independent of fixation condition.

## GENERAL DISCUSSION

Our findings from these four studies suggest that voluntary attentional cues benefit voluntary eye movements significantly greater than reflexive eye movements. In the voluntary task, response times were always greatly facilitated and errors significantly reduced when a cue was present. However, voluntary spatial attention only significantly affected reflexive saccade latencies in some conditions (Experiment 2). Endogenous cues indicating the target location for an upcoming eye movement provided absolutely no benefit for target-elicited saccades, unless these cues were randomly intermixed with uncued or neutral trials. It is somewhat surprising that when presented in a pure block of trials where the endogenous cue was 100% predictive of target location, subjects could not use these cues to improve the efficiency of target-elicited saccades. This occurred despite the fact that the same cues consistently improved performance (both latencies and errors) of antisaccades, whether they were presented in a pure block of trials or mixed with uncued or neutral trials. Thus, there is a striking dissociation in the effects of voluntary spatial attention on these two different types of eye movements. The remainder of the discussion will consider possible explanations and implications of these findings.

Our primary finding was that endogenous, symbolic (arrow) cues presented at fixation facilitated the execution of voluntary eye movements, specifically antisaccades. This finding is consistent with previous claims that saccade latencies can be reduced by voluntary allocation of spatial attention to the location for an upcoming saccade (e.g., [1,2]). Shepherd et al.[1] took the issue further, arguing that execution of an eye movement in the absence of peripheral stimulation could not occur without a corresponding shift of spatial attention. Such an attentional shift would certainly be required to perform the antisaccade task in this experiment.

This facilitative effect of voluntary attention on voluntary eye movements is consistent with theoretical positions assuming an explicit link between the neural systems governing spatial attention and those governing saccadic eye movements (e.g., [3,4,13,22]). Our study focused on the likelihood that voluntary and reflexive eye movements are controlled by physiologically distinct neural systems, which may be differentially affected by the neural system governing voluntary attention. While there were striking voluntary attentional effects on the execution of antisaccades, the influence of voluntary attention on reflexive saccades was not as obvious. Indeed, unless attentional cued trials were intermixed with uncued or neutral trials (i.e., Experiments 2 and 3), voluntary spatial attention had no influence on the execution of target-elicited saccades.

Perhaps voluntary cues failed to facilitate reflexive saccades because of the fast nature of their execution (floor effect). Indeed, overall latencies for reflexive saccades were faster than those for antisaccades. However, it is unlikely that cues failed to influence saccadic latencies because of a floor effect for two reasons. First, the mixed vs. blocked cue manipulation clearly had an influence on cueing effects for reflexive saccades, despite having no overall effect on response time. Second, a comparison of Experiments 2 and 3 shows a clear benefit from alerting properties of the attentional cue. When alerting cues are free to influence response latency (e.g., Experiment 2), the cued-prosaccade condition is faster than the uncued-prosaccade condition. Only when alerting effects are controlled (Experiment 3) do cues fail to affect the latencies of reflexive saccades so drastically. Both the effect of mixing cued and uncued trials, and the influence of alerting effects, demonstrate that latencies for reflexive saccades can vary under the testing conditions used. In addition, and perhaps most relevant to the present study, previous studies have shown that *reflexive* spatial attention, in the form of a noninformative peripheral spatial cue, will significantly facilitate a prosaccade to a target beginning as early as 67 ms after cue onset[28,29]. Hence, *reflexive* spatial attentional cues (unlike the voluntary spatial cues used in the present study) are also able to reduce prosaccade latency. If there truly was a floor effect, none of these manipulations should have been able to reduce prosaccade latency.

It is possible that the weak or absent voluntary attentional effects on reflexive saccades compared to voluntary saccades suggest something about the relationship between voluntary attention and reflexive eye movements. The present data imply the systems governing voluntary attention have a more direct influence on voluntary saccades as compared to reflexive saccades. Converging evidence for this conclusion comes from a study by Stuyven et al.[30] using a dual-task paradigm. Stuyven et al. required subjects to perform voluntary or reflexive saccades either by themselves or while engaging in a secondary task. They found that a secondary, attention-demanding task interfered with the execution of voluntary eye movements. However, interference effects were minimal when a purely reflexive saccade task was used. This is consistent with the view that either the planning or execution of voluntary eye movements requires the use of a general-purpose executive system that is not needed for eye movements controlled by exogenous stimulus events. This executive system may be utilized for a variety of cognitively demanding processes, including the voluntary allocation of visual attention.

The precise locus of this executive system is unclear, but possibly involves areas believed to be important in working memory (i.e., DLPFC). There is a significant amount of evidence indicating that individuals with various disorders that demonstrate attentional deficiencies have a great deal of difficulty with eye movements requiring some degree of voluntary control, but no apparent difficulty with more reflexive saccades. For example, schizophrenic patients typically show poor performance on antisaccade and voluntary attention tasks, but do not differ from controls on tasks involving reflexive saccades[31,32,33,34]. Similarly, individuals with Parkinson's disease, who are also often characterized as suffering from attentional abnormalities, are deficient on a variety of voluntary saccade paradigms, but show no deficit or better performance on reflexive saccades than normal individuals[35,36]. In fact, for a number of quite different disorders, poor performance on voluntary saccades seems to be associated with better (or hyper-) reflexive performance (e.g., in patients with frontal eye field lesions[16]; in schizophrenic patients[37]; in Parkinson's disease patients[35,36]; and, in normals subjects with high schizotypy[38]). The results of these studies agree with Sereno's tonic inhibition model[22], where the voluntary system plays a role in the tonic inhibition of the reflexive system. However, in the present study, we find no evidence that voluntary attention inhibits a reflexive saccade.

The present study also consistently found that cueing effects were larger when the attentional cues were intermixed with no cue (Experiment 2) or neutrally cued trials (Experiment 3). Furthermore, mixing cued and neutral or uncued trials created a cueing effect for reflexive saccades that was not present under blocked presentation (Experiments 2 and 3). Thus, as expected, the mixed presentation did increase the effectiveness of the attentional cues. It has previously been shown that latencies for voluntary saccades can be modulated by varying the degree of attention allocated to performance of the eye movements[30]. The observation of increased cueing effects under mixed presentation was perhaps the result of increasing the salience of the cues. Presumably, these more salient cues elicited more voluntary attention when intermixed with no cue or neutral trials, and thus produced bigger effects.

Unlike Fischer et al.[6], in Experiment 4 we found that voluntary attention did not produce inhibition of prosaccade latencies in any of the three fixation conditions, including the gap paradigm. Voluntary spatial attention appeared to affect both prosaccades and antisaccades independently of fixation condition. That is, under all three fixation conditions, voluntary attention facilitated and reduced errors for voluntary saccades, but it had no significant facilitatory or inhibitory effects on the response times for prosaccades. Fischer and Breitmeyer[6] tested subjects using only overlap and gap paradigms, finding inhibition in the gap but not the overlap. Specifically, Fischer and Weber[39] suggest that engaged visual attention tends to eliminate the express saccades as well as the fast regular saccades. However, the present study found that the step paradigm had the weakest cueing effects compared to gap or overlap in all cases. Interestingly, Sereno[26] used a step paradigm. Thus, it seems that if inhibition were to occur in the present design, it would have been in the step paradigm and not the gap.

Recent evidence from Ristic et al.[40] provides a possible explanation for the discrepancy between our findings and Fischer's[6]. Ristic[40] asserts that directional cues, such as an arrow cue used in the present study, overestimate the contribution of volitional processes because these cues themselves elicit a reflexive response. That is, Ristic consistently found that cueing effects were significantly larger when directional cues such as an arrow were used rather than purely symbolic, nondirectional cues (such as a number). Fischer and Breitmeyer[6] used a nondirectional cueing system, whereas the present study used directional cues. Hence, if an arrow cue indeed elicits a reflexive attentional response, this reflexive facilitation might counter any inhibition that arises from its voluntary attentional effects and hence result in a lack of inhibition seen in the present study.

## CONCLUSIONS

It is clear from these data that reflexive (i.e., target elicited) eye movements benefit from voluntary spatial attention to a far lesser degree than do voluntary eye movements. In fact, under blocked cue presentation target-elicited eye movements appear to be largely independent of any attentional influence at all. Both the present data and converging evidence from other sources suggest that executive attentional processes are crucially important in control of voluntary or internally generated eye movements, but play a diminishing role with respect to reflexive saccades.

Because of the clearly different effects of voluntary spatial attention on voluntary and reflexive saccades, attentional and eye movement systems should not be considered homogenous entities. Hence, in order to answer the question, "Does attention facilitate or hinder eye movements?" at least two prior questions should be addressed. First, what type of attention is one speaking about? Voluntary spatial attention may involve different neural systems than reflexive spatial attention. Second, what type of eye movements are required? The data from the present study strongly suggest that voluntary spatial attention greatly benefits voluntary eye movements, but has minimal effects on reflexive, target-elicited, eye movements.

## ACKNOWLEDGMENTS

This work was supported in part by grants from the James S. McDonnell Foundation, NARSAD, the Summer Research Program at the University of Texas – Houston, and NIH grants (R01 MH63340 and R01 MH65492).
